# Estimation of Aboveground Biomass in Alpine Forests: A Semi-Empirical Approach Considering Canopy Transparency Derived from Airborne LiDAR Data

**DOI:** 10.3390/s110100278

**Published:** 2010-12-29

**Authors:** Andreas Jochem, Markus Hollaus, Martin Rutzinger, Bernhard Höfle

**Affiliations:** 1 alpS-Centre for Climate Change Adaptation Technologies, Grabenweg 3, 6020 Innsbruck, Austria; 2 University of Innsbruck, Department of Geography, Innrain 52, 6020 Innsbruck, Austria; E-Mail: martin.rutzinger@uibk.ac.at; 3 Vienna University of Technology, Institute of Photogrammetry and Remote Sensing, 1040 Vienna, Austria; E-Mail: mh@ipf.tuwien.ac.at; 4 Faculty of Geo-Information Science and Earth Observation of the University of Twente (ITC), 7500 Enschede, The Netherlands; 5 University of Heidelberg, Department of Geography, 69120 Heidelberg, Germany; E-Mail: bernhard.hoefle@geog.uni-heidelberg.de

**Keywords:** airborne LiDAR, biomass, semi-empirical model, 3D point cloud, linear regression

## Abstract

In this study, a semi-empirical model that was originally developed for stem volume estimation is used for aboveground biomass (AGB) estimation of a spruce dominated alpine forest. The reference AGB of the available sample plots is calculated from forest inventory data by means of biomass expansion factors. Furthermore, the semi-empirical model is extended by three different canopy transparency parameters derived from airborne LiDAR data. These parameters have not been considered for stem volume estimation until now and are introduced in order to investigate the behavior of the model concerning AGB estimation. The developed additional input parameters are based on the assumption that transparency of vegetation can bemeasured by determining the penetration of the laser beams through the canopy. These parameters are calculated for every single point within the 3D point cloud in order to consider the varying properties of the vegetation in an appropriate way. Exploratory Data Analysis (EDA) is performed to evaluate the influence of the additional LiDAR derived canopy transparency parameters for AGB estimation. The study is carried out in a 560 km^2^ alpine area in Austria, where reference forest inventory data and LiDAR data are available. The investigations show that the introduction of the canopy transparency parameters does not change the results significantly according to R^2^ (R^2^ = 0.70 to R^2^ = 0.71) in comparison to the results derived from, the semi-empirical model, which was originally developed for stem volume estimation.

## 1. Introduction

In times of higher market prices of fossil fuels and due to the increasing environmental and economic threats of climate change, there will be a rising demand for renewable energy production, such as solar or bio energy. The latter is the focus of the presented paper. Accurate estimation of Aboveground Biomass (AGB), also referred to as dry total tree biomass, in forested areas is essential for developing sustainable low carbon climate friendly strategies. This includes the reduction of costs for the provision of energy resources, the mobilization of wood in local forests and the optimization of timber harvesting chains in order to minimize the environmental impact. AGB is defined as the total amount of aboveground oven dry mass of a tree, which is expressed in tons per unit area [1]. It can be directly converted to the total carbon content that is stored in a forest. Having knowledge about the spatial distribution of the carbon content is important in understanding the carbon cycle [2].

In contrast to time consuming and expensive field methods remote sensing such as spaceborne optical remote sensing or synthetic aperture radar (SAR) is capable for mapping area-wide forest inventory (FI) data in a cost effective, fast and accurate way and has been used widely to retrieve AGB [3–5]. A review of the latest developments in the different fields of remote sensing for forest biomass assessment is given in Koch [6]. Remote sensing based estimates of AGB are mostly based on relationships between reference biomass and various pixel values indicating, e.g., reflectance, greenness of vegetation and/or brightness temperature [7]. However, such methods require an extensive set of reference AGB that can be derived by two major ways: (i) tree specific functions estimating biomass directly from individual tree measurements such as diameter at breast height (DBH), tree height (H), crown length (CL) and/or crown width (CW) [8,9] or (ii) tree specific biomass expansion factors transforming stem volume into AGB [10,11], whereas stem volume is estimated from DBH and H as described in, e.g., Hollaus [12].

In recent years Airborne Laser Scanning (ALS), also referred to as Light Detection and Ranging (LiDAR), has been established as a standard technology for high precision three dimensional topographic data acquisition. The three dimensional information is obtained by using an ALS system, which consists of three main components: (i) a Global Positioning System, which is used to record the aircraft position, (ii) an Inertial Measurement Unit (IMU) that measures the angular attitude of the aircraft (roll, pitch and heading), and (iii) a laser scanner unit transmitting short and collimated pulses towards the Earth surface and recording both the travel time of the laser beam and the energy (intensity), which is scattered by the target surface [13]. By taking the measurements of the GPS/IMU and the travel time of the laser beam into account, the coordinates of the vegetation and terrain scatterers can be determined with high accuracy in a suited georeferenced coordinate system [14]. The obtained geometrical information is stored in a 3D point cloud (x,y,z), whereas each point is tagged with auxiliary information such as strength of backscatter and scan angle. In contrast to conventional sensors (e.g., passive optical), LiDAR is less sensitive to cloud cover and shadows and is able to penetrate the vegetation canopy through gaps between leaves and branches. Thus, LiDAR data represents the full three dimensional structure of the forest canopy and has been adopted as a fast and accurate indirect measure for AGB quantification [15–22]. LiDAR based estimation of AGB can be performed either on individual tree level [23] or on regional level [15,24]. Approaches estimating AGB at individual tree level require high point densities (>5 points/m^2^) and are mostly based on regression models focusing on a relationship between LiDAR derived individual tree parameters (e.g., tree height, crown dimensions) and field based estimates of AGB. Area-wide AGB estimation on regional level can also be performed with low point density LiDAR data and is mainly based on the extrapolation of FI reference data measured at stand or plot level. Therefore, the vertical distribution of the laser echoes is analyzed at stand or plot level in order to derive various statistical quantities that are used as input parameters for empirical models estimating area-based forest inventory parameters (e.g., mean tree height, basal area, stem volume) and AGB, respectively. Both approaches are mainly based on the geometrical information of the point cloud. The usage of the intensity information of the LiDAR data as a complimentary data source offers promising opportunities for, e.g., tree species classification [25–28], which could enhance the AGB estimations [29]. However, this requires an appropriate calibration of the data as described in, e.g., Höfle and Pfeifer [13]. Current methods estimating FI data and AGB, respectively, on regional level with LiDAR data mainly involve the use of empirical models by using linear or nonlinear regression analysis. Such models work reliably in areas of flat terrain and in tree plantations. In mountainous regions as well as in mixed and multi-story forested areas the derivation of FI variables is still a matter of research [12,30,31]. Hollaus *et al.* [32] developed a semi-empirical model for stem volume estimation and applied it to a 128 km^2^ alpine forest. The model was evaluated by comparing it to the multiplicative empirical model of Naesset [33]. For the investigated alpine area both models showed promising results and reached high coefficients of determination (R^2^ = 0.76 – 0.86). Furthermore, the model was successfully applied for the entire Federal State of Vorarlberg, Austria with an area of 2,601 km [34]. In contrast to empirical models, semi-empirical models rely partly on physical assumptions and empirical measurements. By using such models an interpretation of the model parameters might be possible because only input parameters of the same physical units are used and the logical connection between the target variable and LiDAR data is respected [34].

In this paper the semi-empirical model of Hollaus *et al.* [32] is investigated concerning its reliability for area-wide AGB estimation of a 560 km^2^ alpine area. Furthermore, the model is extended by different canopy transparency parameters (CTPs) derived from LiDAR data in order to consider the varying properties of vegetation within the study area. These parameters are based on the assumption that transparency of vegetation can be measured by determining their penetration of the laser light through the canopy. The effect of the integrated CTPs is evaluated by comparison with the results of the model not explicitly considering the transparency of vegetation. An Exploratory Data Analysis (EDA) is performed to investigate the behavior of the different extended models for AGB estimation.

## 2. Study Area and Data

### 2.1. Study Area

The investigated alpine spruce dominated forest land is located in the southern part of the Federal State of Vorarlberg (Austria) in the so-called *Montafon* region and covers an area of 560 km^2^. The elevations within the area range from 800 m above sea level in the valleys to 3,312 m at the Piz Buin Mountain in the Silvretta Mountain range. The landscape is characterized by coniferous and mixed forests, alpine meadows, alpine wasteland and agricultural land. The average timberline is at about 1,950 m whereas two thirds of the forests are located below 1,000 m. The main tree species in the area are Norway spruce (*Picea abies*) with 96% and fir (*Abies alba*) with 3% [35]. About the half of the forests within the study area are managed by the local forest administration Stand Montafon Forstfonds. A detailed forest inventory is operated by the local forest administration, which is used as reference data for the presented study.

### 2.2. Local Forest Inventory Data

**Figure 1.**
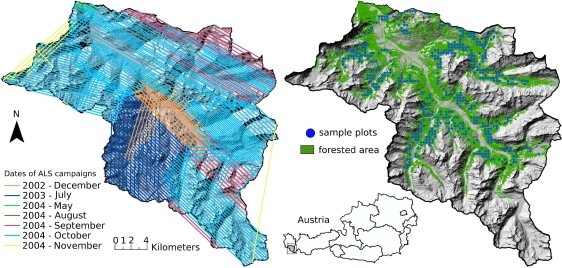
The study area is situated in the western part of the Austrian Alps in the Montafon region. The image on the left shows the dates and the flight paths of the ALS campaigns. The blue circles on the right image represent the location of the forest inventory plots collected by the local forest administration Stand Montafon Forstfonds.

The forest administration Stand Montafon Forstfonds manages about 65 km^2^ of forests in the *Montafon* region. For this study forest inventory (FI) data from 500 sample plots, which are regularly distributed in a 350 m grid are available (Figure 1). They were collected in the year 2002. For each sample plot both the tree specific parameters, such as tree height, tree species and DBH were measured using the angle count sampling method [36]. This measurement approach results in plot areas and number of sampled trees that strongly vary from sample plot to sample plot. For the selection of the trees, a relascope with a relascopic factor of four was used. Further details on measuring and estimating forest inventory parameters (e.g., tree heights, tree coordinates, center coordinates of the sample plots, stem volume) can be found in Hollaus *et al.* [37]. A co-registration of the forest inventory data to the LiDAR data as described in Dorigo *et al.* [38] is required to correct the possible inaccuracies in the spatial positions between the LiDAR and the forest inventory data. For this study 488 of the 500 available sample plots are successfully co-registered to the LiDAR data using the method of Dorigo *et al.* [38].

### 2.3. Determination of Reference AGB

In this study AGB per unit area is used as ground reference quantity. It is estimated from stem volume by means of tree specific expansion factors as described in Weiss *et al.* [10]. Stem volume is assessed as described in Hollaus *et al.* [37] for every single tree that was selected according to the angle count method. The used equations are based on a so called form-height concept meaning that stem volume is estimated by transforming the conical shape of a stem to a cylinder, whereas the diameter of the cylinder corresponds to the DBH [37]. The assessed stem volume of each tree is transformed into dry stem biomass by using tree specific average raw density factors [10]. The next step contains the transformation of the dry stem biomass to dry total tree biomass by means of factors described in Körner *et al.* [39], whereas different tree species and age classes lead to different factors as given in Weiss *et al.* [10]. After AGB assessment of single trees, AGB per unit area is calculated for each sample plot with the following formula:

(1) $$\mathrm{AGB}=\sum_{i=1}^{n}\frac{k}{{\left(\frac{\mathrm{DBH}}{2}\right)}^{2}*\pi }*{\mathrm{AGB}}_{i}$$

where AGB is the aboveground biomass per unit area in tons per hectare [t ha^−1^], *k* is the relascopic factor (set to 4), AGB_i_ is the aboveground biomass of a single tree in kilograms and n is the number of measured trees per sample plot unit.

### 2.4. Airborne Laser Scanning Data

The LiDAR data were acquired during several flight campaigns in the framework of a commercial Vorarlberg-wide terrain mapping project using Optech Airborne Laser Terrain Mapper systems (ALTM 1225, ALTM 2050) and a Leica ALS-50 scanner. All campaigns recorded first and last echoes and took place under snow-free conditions in the years 2002 to 2004. The LiDAR data were acquired at an average flying height of 1,100m above ground (Figure 1). The Optech sensors have a beam divergence of 0.3 mrad and the ALS-50 scanner a beam divergence of 0.33 mrad. The beam divergence resulted in a mean footprint diameter of 0.33 m and 0.36 m, respectively for the average flying height. The mean point densities within the study area vary between 0.9 points/m^2^ and 2.7 points/m^2^. Further information about the used LiDAR sensors are listed in Table 1.

The georeferenced 3D point clouds as well as the Digital Terrain Model (DTM) and the Digital Surface Model (DSM) were provided by the Land Survey Administration Feldkirch, Austria. The DTM, which has a spatial resolution of 1 m was generated by using last echoes and applying the hierarchic robust filter technique as described e.g., in Kraus and Pfeifer [40]. A Canopy Height Model (CHM) that is produced by subtracting the DTM from the DSM is used to improve the co-registration of the forest inventory data to the LiDAR data as described in Dorigo *et al.* [38].

## 3. Methodology

### 3.1. Semi-Empirical Model

The semi-empirical model is based on the assumption that AGB, given in tons per hectare (t ha^−1^) can be expressed as a linear function of the canopy volumes (*V*_can_(m^3^ ha^−1^)). The canopy volume is defined as the entire volume between the terrain surface and the topmost tree surface. The calculation of *V*_can_ is based on the heights (*i.e.*, the relative heights to the ground) of the LiDAR first echoes and is performed for different canopy height intervals to consider the variability of the vertical and horizontal structure of the canopy. *V*_can_ is determined by using a fixed circular reference area (*A*(m^2^)) around the center of the forest inventory sample plots. The height above terrain surface of each first echo point is used to classify the points into *m* different height classes, whereas all points having a height value of less than 2.0 m are classified as points reflected from the terrain, bushes, stones *etc.* [33] and are not included into the canopy volume calculation. *A* is split into several sub-areas *A*_i_ (i=1,2...,m), whereas the size of *A_i_* is determined by the relative proportion *p*_fe,*i*_ (between 0 and 1, whereas the sum of *p*_fe,*i*_ is 1) of first echo points, whose heights fall within the canopy height class *i. V*_can,*i*_ is calculated as:

(2) $$V_{\text{can},i}=\frac{A*p_{\text{fe},i}*{\mathrm{ch}}_{\text{mean},i}}{A}=p_{\text{fe},i}*{\mathrm{ch}}_{\text{mean},i}$$

where *ch*_mean,*i*_ is the mean canopy height of all first echoes within the corresponding canopy height class. To guarantee that both, the reference AGB (t ha^−1^) and the estimated AGB are given per unit area, *V*_can,*i*_ has to be divided by *A*. The semi-empirical model estimating AGB from LiDAR data was formulated as

(3) $$\mathrm{AGB}=10^{4}\sum_{i=1}^{m}\beta_{i}*V_{\text{can},i}$$

where *m* is the number of canopy height classes, *β_i_* are the unknown model coefficients estimated with a least squares approach and can be interpreted as the fraction of the corresponding canopy volume to the reference AGB. The factor 10^4^ was added to take the different area units of AGB (t ha^−1^) and *V*_can,*i*_ (m^3^m^−2^) into account.

According to former studies [32,34] four canopy height classes having a canopy height interval of 10 m are used for the calculation of the canopy volume. *V*_can,1_ ranges between 2 m and 12 m, *V*_can,2_ ranges between 12 m and 22 m, *V*_can,3_ ranges between 22 m and 32 m and *V*_can,4_ contains all first echoes having a height greater than 32 m.

### 3.2. Canopy Transparency Parameters

In this study three different CTPs are defined and investigated with respect to their influence on AGB estimation of the semi-empirical model. They describe the transparency of the canopy surface towards the first laser echoes and are introduced in order to describe the varying properties of the vegetation within the study area in more detail. The CTPs underlie the assumption that all laser pulses enter the canopy parallel to the stems of the trees. Due to lack of LiDAR data representing identical canopy structures scanned with various scan angles, the influence of flying altitude and scan angle on the penetration of the laser pulses into the canopy and their impact on the resulting 3D point cloud have not been assessed in this study as performed in e.g., Morsdorf *et al.* [41] and Naesset [42].

The CTPs are integrated in the semi-empirical model of Hollaus *et al.* [32] to reduce *V*_can,*i*_ in areas that are transparent towards the laser beam because it is assumed that such areas contribute less to AGB than areas that are not penetrated by the laser shots. Due to overlapping flight strips, changing airplane attitude and topographic conditions, the distance between points as well as the point density vary between the sample plots. These circumstances are considered in each of the following parameters. Hence, the developed CTPs should guarantee that the estimated AGB of identical sample plots having different point densities is comparable to each other.

#### Canopy Transparency Based on a Static Search Radius

**Figure 2.**
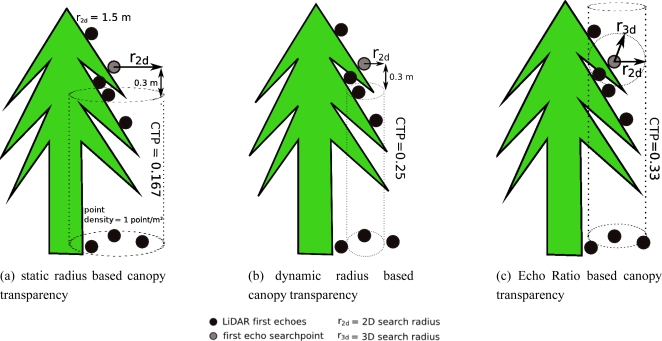
Illustration of the canopy transparency parameters (CTPs), which are applied to every first echo laser point. In **(a)** a static search radius, in **(b)** a dynamic search radius depending on the sample plot first echo point density is used to calculate the canopy transparency towards the laser echoes. The canopy transparency parameter in **(c)** is based on the Echo Ratio (ER).

As illustrated in Figure 2(a), the transparency of the canopy towards the laser echoes of the current location is computed by searching all first echo points (*n*_2d_) within a static search radius *r*_2d_ (e.g., 1.0 m, measured in 2D) that were reflected from below the current search point. The term static search radius means that the same search distance is applied on every single point of each sample plot. The detected points must have a minimum vertical distance of, e.g., 0.3 m to guarantee that points that were reflected from the canopy surface, but differ slightly in elevation due to the sloped canopy surface, are not selected as points that penetrated the canopy surface. However, the varying average first echo point densities (*DP*_fe_) between the different circular sample plots are not considered yet and a normalization of *n*_2d_ with the *DP*_fe_ of the corresponding circular sample plot is required. *DP*_fe_ is determined by dividing the number of first echoes within the corresponding sample plot (*n*_fe_) by its area (*A*). The following equation is used to compute the CTP based on a static search radius (*CTP*_static_).

(4) $${\mathrm{CTP}}_{\text{static}}=\frac{1}{\left(n_{2d}/\frac{n_{\text{fe}}}{A}\right)}=\frac{1}{\left(n_{2d}/{\mathrm{DP}}_{\text{fe}}\right)}$$

where *n*_2d_ is the number of points (including the search point) found in a search distance of *r*_2d_, whereas the height of the vertical search cylinder is equal to the height of the search point minus the defined minimum vertical distance.

#### Canopy Transparency Based on a Dynamic Search Radius

This CTP is based on a dynamic 2D search radius (*CTP*_dynamic_) in order to find all first echoes that were reflected from below the current search point. The selected points must also have a minimum vertical distance of e.g., 0.3 m from the current search point to overcome the problems mentioned above. Dynamic search radius means that *r*_2d_ is adjusted to the *DP*_fe_ of the corresponding sample plot. Hence, it varies between the sample plots but takes the varying *DP*_fe_ between the sample plots into account. *r*_2d_ is defined as:

(5) $$r_{2d}=\sqrt{\frac{A}{n_{\text{fe}}*\pi }}$$

*CTP*_dynamic_ is calculated using the following equation:

(6) $${\mathrm{CTP}}_{\text{dynamic}}=\frac{1}{n_{2d}}$$

#### Canopy Transparency Based on the Echo Ratio

The Echo Ratio (ER), which is a measure for local transparency and canopy surface roughness has been used in various studies to separate solid objects characterized by planarity such as building roofs from non-planar objects like vegetation [43]. In this study the ER value is used as a measure of transparency of vegetation. *CTP*_ER_ is derived for each first echo and is defined as:

(7) $$\mathrm{ER}={\mathrm{CTP}}_{\mathrm{ER}}=\frac{n_{3d}}{n_{2d}}$$

As illustrated in Figure 2(c), *n*_3d_ is defined as the number of first echoes (including the search point) found in a dynamic search distance measured in 3D. *n*_2d_ is the number of first echo points found in the same distance measured in 2D, whereas the vertical expansion of the search cylinder is infinite. The dynamic search distance is calculated according to Equation 5 taking the varying point densities of the sample plots into account. ER decreases from dense (non-transparent) to less dense (transparent) vegetated areas.

### 3.3. Integration of Canopy Transparency Parameters

Each of the LiDAR based CTPs (Section 3.2) is integrated in the semi-empirical AGB model (Section 3.1). This leads to four different semi-empirical models (including the model without a CTP), which are analyzed according to their predictive accuracy. The canopy transparency is calculated for every single first echo point. The integration of *CTP* is performed by altering Equation 2 as:

(8) $$V_{\text{can},i}=p_{\text{fe},i}*\frac{\sum_{k=1}^{n_{\text{fe},i}}{\mathrm{ch}}_{\text{fe},k}*{\mathrm{CTP}}_{k}}{n_{\text{fe},i}}=\frac{n_{\text{fe},i}}{n_{\text{fe}}}*\frac{\sum_{k=1}^{n_{\text{fe},i}}{\mathrm{ch}}_{\text{fe},k}*{\mathrm{CTP}}_{k}}{n_{\text{fe},i}}=\frac{\sum_{k=1}^{n_{\text{fe},i}}{\mathrm{ch}}_{\text{fe},k}*{\mathrm{CTP}}_{k}}{n_{\text{fe}}}$$

where *n*_fe,*i*_ is the number of all first echoes and *ch*_fe,k_ is the height of each first echo point within the corresponding height class *i. n*_fe_ is the total number of all first echoes within *A. CTP*_k_ is the canopy transparency parameter of the corresponding first echo point and is set to one if *V*_can,*i*_ is calculated without any CTP and hence equal to the model as described by Hollaus *et al.* [32].

### 3.4. Calibration and Validation of the Semi-Empirical Model

The estimation of the optimal sample plot area is performed as described in former studies [12,32,34] and is based on the unaltered semi-empirical model using four canopy height classes with a canopy height interval of 10.0 m (Section 3.1). A leave one out cross validation procedure is performed to assess the predictive accuracy of the calibrated model. The LiDAR data were acquired partly under leaf-on as well as under leaf-off conditions (Section 2.4). This could result in different canopy volumes for deciduous trees even if they have similar stem volumes. To avoid different flight dates having an effect on the calibration of the semi-empirical model, coniferous sample plots are separated from deciduous ones by applying a 90% coniferous trees threshold. The selected sample plots are used for the determination of the optimal circular sample plot area. Those reference sample plots, where the sampled trees are outside the estimated sample plot size are excluded for the further calculations. All sample plots fulfilling these conditions are used for estimating and calibrating the *β* coefficients of the semi-empirical models. A comparison with the original model is performed by both their predictive accuracies and by a set of EDA.

## 4. Results and Discussion

### 4.1. Selection of Reference Sample Plots

The 90% coniferous trees threshold resulted in a selection of 450 out of 488 successfully co-registered sample plots. These sample plots are taken as input for the determination of the optimum circular sample plot size. As shown in Table 2 a sample plot radius of 12.0 m results in the highest R^2^ and the lowest SD of the prediction errors and thus in the highest accuracy of the calibrated model.

In a next step, those sample plots, which contain only trees that are located within a sample plot radius of 12.0 m are selected. 196 out of 450 coniferous sample plots fulfill this condition and are taken for the calibration of the semi-empirical models.

### 4.2. Calibration of the Semi-Empirical Model

The models are calibrated using the 196 selected sample plots (Section 4.1). The first echo point cloud serves as input for calculating the canopy volumes.

**Figure 3.**
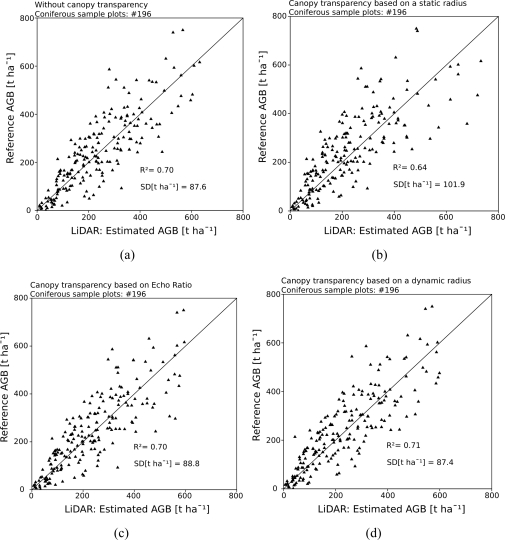
Scatter plots showing the aboveground biomass derived from the local forest inventory versus the aboveground biomass estimated from 3D LiDAR first echo point cloud data. Different canopy transparency parameters (b–d) are introduced and investigated concerning AGB estimation.

Calibrating the model without using a CTP results in a R^2^ of 0.70 and a SD of the prediction errors of 87.6 t ha^−1^ (35.8%). Extending the model by the CTP based on a static radius of 1.0 m degrades the R^2^ to 0.64, while the SD of the prediction errors increases to 101.9 t ha^−1^ (41.7%). Normalizing the number of points found below the current canopy point by the sample plot point density *DP*_fe_ is required (Equation (4)). If *DP*_fe_ is not considered, R^2^ decreases to 0.55, while the SD of the prediction errors increases to 113.7 t ha^−1^ (46.5%). Introducing the CTP based on the ER as a measure for transparency of vegetation towards the laser beams results in a R^2^ of 0.70 and in a SD of the prediction errors of 88.8 t ha^−1^ (36.3%). Extending the model by the CTP based on a dynamic search radius a R^2^ of 0.71 and a SD of the prediction errors of 87.4 t ha^−1^ (35.8%) is achieved. The accuracy statistics, the *β* coefficients of the calibrated models and the p-values of the corresponding input parameters are shown in Table 3. Figure 3 shows the scatter plots of the reference AGB versus the AGB estimated from LiDAR data.

According to R^2^ *CTP*_dynamic_ results in a minor improvement compared to the model not using any canopy transparency factor. The CTP based on a dynamic search radius leads to a slight increase of R^2^ to 0.71. The accuracy of the model using the CTP based on the ER is similar to the accuracy of the model not using any CTP. The R^2^ values in the presented approach differ from the study of Hollaus *et al.* [32]. They achieved R^2^ values up to 0.86 for stem volume estimation. These deviations can be explained by the different target variables of the models (AGB versus stem volume estimation) and the transformation of stem volume to AGB, which is accompanied with uncertainties (Section 2.2), respectively. The *β* coefficients represent the fraction of the AGB occupied by the corresponding canopy height class (Section 3.1). Analyzing these coefficients confirmed the findings of Hollaus *et al.* [32], who stated that canopy heights between 22 m and 32 m are the highest contributors to growing stock. In this study these canopy heights are identified as the highest contributors for AGB estimation. For all semi-empirical models *β*_3_ has the highest fraction for calculating AGB and varies between 29.75 × 10^−4^ and 59.50 × 10^−4^ (Table 3).

The CTPs are introduced in order to reduce *V*_can,*i*_ in areas that are transparent towards the laser beam. It is assumed that such areas contribute less to AGB than areas that are not penetrated by the laser shots. As shown above, the integration of the CTPs has not led to a significant improvement concerning R^2^. This can be explained by the usage of first echoes for the calculation of *V*_can,*i*_. First echoes being reflected from below the canopy surface are characterized by lower heights than laser points being reflected from, e.g., the tree crowns and hence, contribute less to the calculation of *V*_can,*i*_. Therefore, the integration of CTPs that are also based on first echo point clouds may not change the behavior of the semi-empirical model concerning R^2^ significantly. However, the reflection of first echoes from below the canopy surface is also dependent on the settings of the LiDAR sensors acquiring the three dimensional point cloud of the area of investigation such as beam divergence and range between sensor and object (*i.e.*, nominal footprint size). The characteristics of the different LiDAR sensors used during the ALS mapping campaigns (Section 2.4), the impact of the LiDAR scanning angle and the flying altitude on the resulting 3D point cloud as well as their influence on the semi-empirical model have not been investigated in this study but will be in the focus of future research. It is expected that the number of first return points that are reflected from close to the top of the canopy increases with increasing scan angle due to the increased path length through the canopy. This may affect the semi-empirical model and the derivation of the canopy transparency parameters. Investigations on this topic require a very detailed data basis in order to gain reliable knowledge about the influence of the viewing geometry (*i.e.*, flying altitude and scanning angle) on the penetration of each laser pulse into the canopy. This means that at least a subset of the sample plots within the study area has to be scanned with various scan angles and flying altitudes using different LiDAR sensors [42]. Morsdorf *et al.* [41] assessed the influence of flying altitude and scanning angle on the derivation of forestry parameters such as leaf area index, fractional cover and tree height. Their test site was sampled with two nominal flying altitudes, 500 m and 900 m above ground, whereas the overlap of each flight strip was about 50% with each neighboring strip. This allows the investigation of differences of ALS based estimates with respect to varying flying altitude and scanning angle. They found that the derivation of biophysical vegetation properties is much more affected by flying altitude than by scanning angle. This could also be due to the small scan angle of the laser scanning system (±7.15). The results of Disney *et al.* [44] show that the impact of scanning angle towards LiDAR derived canopy height is greater for conifer than for broadleaf forests. This has been investigated by using detailed 3D models in order to simulate the LiDAR response of young conifer and broadleaf forests. This simulation allowed to test the influence of different LiDAR parameters under a range of set-ups usually not possible in practice.

### 4.3. Exploratory Data Analysis

**Figure 4.**
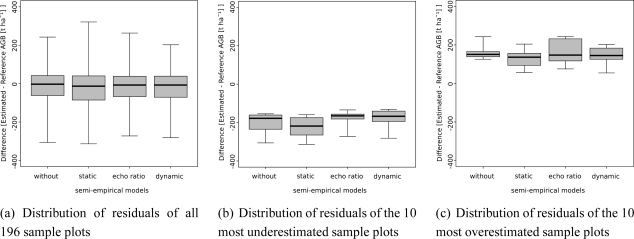
The box-whisker plots show the under- and overestimation of the different semi-empirical models. The reference AGB is subtracted from the AGB estimated from LiDAR data. The impact of LiDAR derived canopy transparency is investigated on sample plots that are highly under- and overestimated by the original model.

Within the EDA all 196 selected sample plots are analyzed according to their under- and overestimation of AGB by the different models. Additionally, the 10 sample plots leading to the highest under- and overestimation, respectively, by the original model are selected for further analysis. This procedure is based on the assumption that the original model leads to outliers concerning AGB estimation due to the heterogeneity of the properties of the vegetation within the study area. Analyzing these sample plots separately offers the possibility to check if the integration of CTPs is useful to consider the varying properties of the vegetation of the outlying sample plots in a proper way. The box-whisker plots in Figure 4 (a−c) indicate the distribution of residuals of the different models. The distribution of all 196 sample plots is shown in Figure 4(a). The introduction of the CTP based on the dynamic search radius leads to a box-whisker plot whose minimum-maximum range is slightly smaller than the range of the box-whisker plot resulting from the model not using a CTP (Figure 4(a)). The model without any CTP leads to values concerning under- and overestimation ranging from −305.91 t ha^−1^ to 242.50 t ha^−1^. The model based on a dynamic search radius results in values ranging from −281.50 t ha^−1^ to 202.00 t ha^−1^. The median value changes from −2.78 t ha^−1^ to −7.30 t ha^−1^ meaning that a higher amount of sample plots is underestimated by using the model based on a dynamic search radius (103 sample plots *versus* 108 sample plots). The CTP based on a static search radius does not lead to any improvement at all. It degrades the results, which can also be concluded from the R^2^ value in Section 4.2. In Figure 4(b,c) the distribution of residuals of sample plots that are strongly under- and overestimated by the original model are analyzed. Underestimation can be compensated using either the CTP based on the ER or on a dynamic search radius, whereas the density parameter based on the ER has the most significant effect. Compared to the original model both density parameters lead to an increase of the minimum, maximum, median and the values of the interquartile. The CTP based on a static search radius does not lead to any improvement of sample plots being strongly underestimated by the original model and leads to a decrease of these values. In Figure 4(c) strongly overestimated sample plots are compared to each other. In this case the CTP based on a static search radius shows the best results and is able to minimize the overestimations. The values of the interquartile as well as the minimum and maximum values decrease. A decrease of the median, minimum and maximum values can also be observed for the distribution based on the model using a dynamic search radius, whereas the value of the third quartile increases. The model using the CTP based on the ER degrades the results concerning AGB estimation of the 10 sample plots selected for investigation in Figure 4(c).

**Figure 5.**
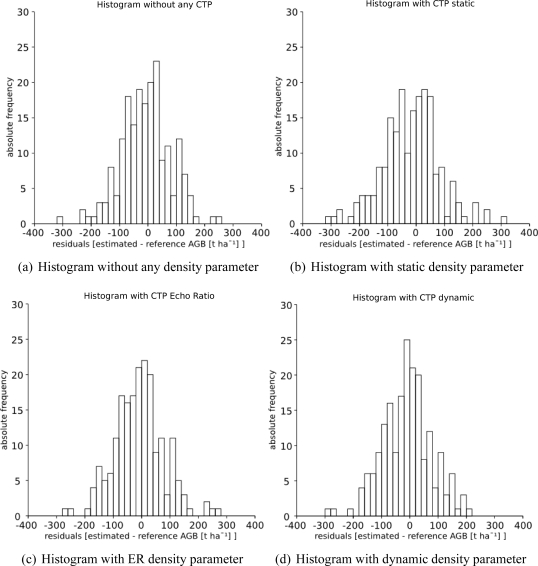
The histograms show the frequency distribution of residuals (estimated minus reference AGB) of all 196 sample plots for all investigated models.

Single outliers of each model can be detected by analyzing the frequency distribution of the residuals in Figure 5. Besides the frequency distribution based on the model using a static search radius the histograms in Figure 5 look very similar. This confirms the robustness of the original model against different canopy surface transparencies. Significant changes can not be observed, neither by an increase of R^2^ nor by a different frequency distribution of the residuals.

## 5. Summary and Conclusions

In this study LiDAR data is used for area-wide AGB estimation of a spruce dominated alpine forest. In the presented approach a semi-empirical model, which was originally developed for stem volume estimation is used and investigated concerning its reliability for AGB estimation. Local forest inventory data are used for the calculation of reference AGB per sample plot by means biomass expansion factors. Furthermore, the semi-empirical model is extended by different CTPs derived from airborne LiDAR data that have not been considered yet and are introduced in order to investigate the behavior of the different models concerning AGB estimation. The introduction of these parameters is based on the assumption that the varying properties of vegetation within the study area can be described in a better way and consequently leads to better result concerning R^2^. The determination of the optimum sample plot size is performed as described in Hollaus [12] and results in a fixed sample plot radius of 12 m. A 90% coniferous trees threshold is applied on each sample plot to avoid deciduous trees having an effect on the calibrated model when LiDAR data are acquired under leaf-on and under leaf-off conditions, respectively. Furthermore, those sample plots that do not contain all sampled trees within the radius of 12.0 m are excluded and are not taken for the calibration of the linear regression models. The determination of the optimal sample plot size as well as the 90% coniferous trees threshold is only applied on sample plots, whose positional accuracies could be improved successfully by applying a co-registration approach [38]. 196 out of 500 sample plots fulfill the aforementioned conditions and serve as reference data.

The results of the presented approach show that the semi-empirical stem volume model can also be used for AGB estimation of a spruce dominated alpine forest. The extension of the model by different CTPs does not change R^2^ significantly. The varying point densities of the sample plots, which are a consequence of overlapping flight strips and the topographic conditions in the Montafon region are considered in each of the presented CTPs, either by adjusting the search radius or by normalizing the number of selected points by the local point density. In future studies the different models will be applied on areas, which are characterized by both a wider range of tree species and a higher point density than it was the case in the presented study. Furthermore, those areas that are strongly over- and underestimated by the original model will be investigated according to their vegetation characteristics in order to use models based on a CTP in such areas. Additionally, the impact of the different LiDAR parameters discussed in Section 4.2 on the resulting 3D point cloud and the semi-empirical model, respectively, have not been considered in this study but will be in the focus of future research.

## Figures and Tables

**Table 1.** Summary of characteristics of applied LiDAR sensors.

	**Sensors**		
**Sensor characteristics**	**Optech ALTM 1225**	**Optech ALTM 2050**	**Leica ALS-50**
Beam Divergence [mrad]	0.3	0.3	0.33
Fields of View [°]	0–40	0–40	up to 75
Wavelength [nm]	1,064	1,067	1,064
Pulse Repetition[kHz]	<25	<50	<83
Multiple Targets	up to 2	up to 2	up to 4

**Table 2.** Determination of the optimum circular sample plot size by analyzing various radii according to their R^2^ and standard deviation of the residuals.

**Sample plot radius [m]**	**8.0**	**10.0**	**12.0**	**14.0**	**16.0**
*R*^2^	0.60	0.64	0.66	0.64	0.61
*SD*[t^3^ ha^−1^]	120.2	111.4	109.0	111.2	115.7

**Table 3.** Accuracy statistics of the fitted AGB models. R^2^, SD of the prediction errors and the estimated *β* coefficients with their corresponding p-values (from t-test) are shown.

Parameters	without CTP	static CTP	EchoRatio CTP	dynamic CTP
*R*^2^	0.70	0.64	0.70	0.71
*SD*[t ha^−1^]	87.6 (35.8%)	101.9 (41.7%)	88.8 (36.3%)	87.4 (35.8%)
*β*1 / p	7.71 × 10^−4^ / 0.15	12.50 × 10^−4^ / 0.21	9.14 × 10^−4^ / 0.365	16.21 × 10^−4^ / 0.05
*β*2 / p	19.91 × 10^−4^ / 1.41 × 10^−12^	37.74 × 10^−4^ / 7.27 × 10^−11^	46.16 × 10^−4^ / 3.34 × 10^−15^	39.02 × 10^−4^ / <2 × 10^−16^
*β*3 / p	29.75 × 10^−4^ / <2 × 10^−16^	54.60 × 10^−4^ / <2 × 10^−16^	59.50 × 10^−4^ / <2 × 10^−16^	50.72 × 10^−4^ / <2 × 10^−16^
*β*4 / p	15.87 × 10^−4^ / 2.15 × 10^−5^	20.23 × 10^−4^ / 0.026	38.12 × 10^−4^ / 1.27 × 10^−6^	24.78 × 10^−4^ / 2.74 × 10^−4^

## References

[1] Brown S. (1997). Estimating biomass and biomass change in tropical forests: A primer. FAO Forestry Paper.

[2] Houghton R.A. (2005). Aboveground forest biomass and the global carbon balance. Glob. Change Biol.

[3] Solberg S., Astrup R., Gobakken T., Naesset E., Weydahl D.J. (2010). Estimating spruce and pine biomass with interferometric X-band SAR. Remote Sens. Environ.

[4] Anaya J., Chuvieco E., Palacios-Orueta A. (2009). Aboveground biomass assessment in Colombia: A remote sensing approach. Forest Ecol. Manage.

[5] Zheng D., Rademacher J., Chen J.C.T., Bresee M., LeMoine J., Ryu S. (2004). Estimating aboveground biomass using Landsat 7 ETM + data a across managed landscape in northern Wisconsin, USA. Remote Sens. Environ.

[6] Koch B. (2010). Status and future of laser scanning, next term synthetic aperture radar and hyperspectral remote sensing data for forest biomass assessment. ISPRS J. Photogramm.

[7] Lin Y., Jaakola A., Hyyppä J., Kaartinen H. (2010). From TLS to VLS: Biomass estimation at individual tree level. Remote Sens.

[8] Ledermann T., Neumann M. (2006). Biomass equations from data of old long-term experimental plots. Austria. J. For. Sci.

[9] Gschwantner T., Schadauer K. (2006). Branch biomass functions for broadleaved tree species in Austria. Austria. J. For. Sci.

[10] Weiss P., Schieler K., Schadauer K., Radunsky K., Englisch M. (2000). Die Kohlenstoffbilanz des Österreichischen Waldes und Betrachtungen zum Kyoto-Protokoll.

[11] Cháidez J. (2009). Allometric equations and expansion factors for tropical try forest trees of eastern Sinaloa, Mexico. Trop. Subtrop. Agroecosyst.

[12] Hollaus M. (2006). Large Scale Applications of Airborne Laser Scanning for a Complex Mountainous Environment.

[13] Höfle B., Pfeifer N. (2007). Correction of laser scanning intensity data: Data and model-driven approaches. ISPRS J. Photogramm.

[14] Kraus K (2007). Photogrammetry: Geometry from Images and Laser Scans.

[15] Lim K., Treitz P. (2004). Estimation of above ground forest biomass from airborne discrete return laser scanner data using canopy-based quantile estimators. Scand. J. Forest Res.

[16] Bortolot Z.J., Wynn R.H. (2005). Estimating forest biomass in small footprint LiDAR data: An individual tree-based approach that incorporates training data. ISPRS J. Photogramm.

[17] Zhao K., Popescu S., Nelson R. (2009). Lidar remote sensing of forest biomass: A scale-invariant estimation approach using airborne lasers. Remote Sens. Environ.

[18] Nelson R., Krabill W., Tonelli J. (1988). Estimating forest biomass and volume using airborne laser data. Remote Sens. Environ.

[19] Lefsky M., Cohen W., Harding D., Parker G., Acker S., Gower S. (2001). Remote sensing of aboveground biomass in three biomes. Int. Arch. Photogram. Rem. Sens. Spatial. Inform. Sci.

[20] Lim K., Treitz P., Baldwin K., Morrison I., Green J. (2003). Lidar remote sensing of biophysical properties of tolerant northern hardwood forests. Can. J. Rem. Sens.

[21] Popescu S., Wynne R., Nelson R. (2003). Measuring individual tree crown diameter with lidar and assessing its influence on estimating forest volume and biomass. Can. J. Rem. Sens.

[22] Nelson R., Short A., Valenti M. (2004). Measuring biomass and carbon in Delaware using an airborne profiling LIDAR. Scand. J. Forest Res.

[23] Popescu S. (2007). Estimating biomass of individual pine trees using airborne LiDAR. Biomass Bioenerg.

[24] Næsset E. (2004). Estimation of above- and below-ground biomass in boreal forest ecosystems. Int. Arch. Photogram. Rem. Sens. Spatial. Inform. Sci.

[25] Brandtberg T. (2007). Classifying individual tree species under leaf-off and leaf-on conditions using airborne lidar. ISPRS J. Photogramm.

[26] Höfle B., Hollaus M., Lehner H., Pfeifer N., Wagner W. Area-based parameterization of forest structure using full-waveform airborne laser scanning data.

[27] Hollaus M., Mücke W., Höfle B., Dorigo W., Pfeifer N., Wagner W., Bauerhansl C., Regner B. Tree species classification based on full-waveform airborne laser scanning data.

[28] Kim S., McGaughey R.J., Andersen H.E., Schreuder G. (2009). Tree species differentiation using intensity data derived from leaf-on and leaf-off airborne laser scanner data. Remote Sens. Environ.

[29] García M., Riaño D., Chuvieco E., Mark Danson F. (2010). Estimating biomass carbon stocks for a Mediterranean forest in central Spain using LiDAR height and intensity data. Remote Sens. Environ.

[30] Maas H.G. (2010). Airborne and Terrestrial Laser Scanning.

[31] Hyyppä J., Hyyppä H., Xiaowei Y., Kaartinen H., Kukko A., Holopainen M (2009). Topographic Laser Ranging and Scanning: Principles and Processing.

[32] Hollaus M., Wagner W., Schadauer K., Maier B., Gabler K. (2009). Growing stock estimation for alpine forests in Austria: A robust LiDAR-based approach. Can. J. Forest Res.

[33] Næsset E. (2004). Practical large-scale forest stand inventory using a small foot print airborne scanning laser. Scand. J. Forest Res.

[34] Hollaus M., Dorigo W., Wagner W., Schadauer K., Höfle B., Maier B. (2009). Operational wide-area stem volume estimation based on airborne laser scanning and national forest inventory data. Int. J. Remote Sens.

[35] Stand Montafon Forstfonds.

[36] Bitterlich W. (1948). Die Winkelzählprobe. Allgemeine Forst- und Holzwirtschaftliche Zeitung.

[37] Hollaus M., Wagner W., Maier B., Schadauer K. (2007). Airborne laser scanning of forest stem volume in a mountainous environment. Sensors.

[38] Dorigo W., Hollaus M., Schadauer K., Wagner W. (2009). An application-oriented automated approach for co-registration of forest inventory and airborne laser scanning data. Int. J. Remote Sens.

[39] Körner C., Schilcher B., Peláez-Riedl S. (1993). Vegetation und treibhausproblematik: Eine beurteilung der situation in österreich unter vesonderer berücksichtigung der kohlenstoff-bilanz. Anthropogene Klimaänderungen: Mögliche Auswirkungen auf Österreich mögliche Massnahmen in Österreich.

[40] Kraus K., Pfeifer N. (1998). Determination of terrain models in wooded areas with airborne laser scanner data. ISPRS J. Photogramm.

[41] Morsdorf F., Frey O., Meier E., Itten K.I., Allgöwer B. (2008). Assessment of the influence of flying altitude and scan angle on biophysical vegetation products derived from airborne laser scanning. Int. J. Remote Sens.

[42] Naesset E. (2009). Effects of different sensors, flying altitudes, and pulse repetition frequencies on forest canopy metrics and biophysical stand properties derived from small-footprint airborne laser data. Remote Sens. Environ.

[43] Höfle B., Mücke W., Dutter M., Rutzinger M., Dorninger P. Detection of building regions using airborne lidar—A new combination of raster and point cloud based GIS methods.

[44] Disney M., Kalogirou V., Lewis P., Prieto-Blanco A., Hancock S., Pfeifer M. (2010). Simulating the impact of discrete-return lidar system and survey characteristics over young conifer and broadleaf forests. Remote Sens. Environ.

